# Demographic differences in and correlates of perceived body image discrepancy among urban adolescent girls: a cross-sectional study

**DOI:** 10.1186/s12887-017-0952-3

**Published:** 2017-12-06

**Authors:** Lorraine B. Robbins, Jiying Ling, Kenneth Resnicow

**Affiliations:** 1 College of Nursing, Michigan State University, 1355 Bogue Street, East Lansing, MI 48824 USA; 2 School of Public Health, University of Michigan, 1415 Washington Heights, Ann Arbor, MI 48109 USA

**Keywords:** Physical activity, Fitness, Adolescents, Females, School, Physical appearance, Perception

## Abstract

**Background:**

Understanding factors related to girls’ body image discrepancy, which is the difference between self-perceived current or actual and ideal body size, is important for addressing body-related issues and preventing adverse sequelae. Two aims were to: 1) examine demographic differences in body image discrepancy; and 2) determine the association of body image discrepancy with weight status, percent body fat, physical activity, sedentary behavior, and cardiovascular (CV) fitness among young adolescent girls.

**Methods:**

The cross-sectional study included a secondary analysis of baseline data from a group randomized controlled trial including 1519 5th–8th grade girls in 24 U.S. schools. Girls completed physical activity and sedentary behavior surveys. To indicate perceived current/actual and ideal body image, girls selected from nine body figures the one that represented how they look now and another showing how they want to look. Girls wore accelerometers measuring physical activity. Height, weight, and percent body fat were assessed. The Progressive Aerobic CV Endurance Run was used to estimate CV fitness. Independent t-test, one- and two-way ANOVA, correlational analyses, and hierarchical linear regressions were performed.

**Results:**

The majority (67.5%; *n* = 1023) chose a smaller ideal than current/actual figure. White girls had higher body image discrepancy than Black girls (*p* = .035). Body image discrepancy increased with increasing weight status (F_3,1506_ = 171.32, *p* < .001). Moderate-to-vigorous physical activity (MVPA) and vigorous physical activity were negatively correlated with body image discrepancy (*r* = −.10, *p* < .001; *r* = −.14, *p* < .001, respectively), but correlations were not significant after adjusting for race and body mass index (BMI), respectively. Body image discrepancy was moderately correlated with CV fitness (*r* = −.55, *p* < .001). After adjusting for demographics, percent body fat, but not CV fitness or MVPA, influenced body image discrepancy. Girls with higher percent body fat had higher body image discrepancy (*p* < .001).

**Conclusion:**

This study provided important information to guide interventions for promoting a positive body image among girls.

**Trial registration:**

ClinicalTrials.gov Identifier NCT01503333, registration date: January 4, 2012.

## Background

Although 32.0% of high school girls who completed the 2015 U.S. Youth Risk Behavior Survey were actually overweight or obese, 60.6% reported trying to lose weight [1]. Consistent with this finding, Duchesne and colleagues [2] noted that as high as 63.5% of adolescent girls experience poor body image. One factor contributing to poor body image is that girls learn from their families, friends, and other sources, such as the media, that thinness is desirable [3]. Of concern is that girls’ negative perceptions of their body weight, shape, or size places them at high risk for low self-esteem, decreased self-worth, poor self-concept, and negative affect [4] or an eating disorder [5, 6]. In contrast, a positive body image may protect against the development of various mental health conditions, such as depression [7].

Body image is widely referred to as the personal internal view or representation [8] or self-evaluation of outer physical appearance [9]. This multi-dimensional construct is often assessed by measures of body satisfaction or esteem and weight satisfaction [9]. Body satisfaction is usually measured in adolescents by asking them to rate their level of satisfaction with aspects of their body, such as height, weight, shape, waist, build, face, and specific body parts [5, 10–13]. Body esteem is relatively similar to body satisfaction, but reflects the level of agreement with positive versus negative aspects related to one’s body (e.g., being proud of one’s body) [9]. Weight satisfaction, commonly referred to as body image discrepancy, is easily measured and defined as the difference between self-perceived current or actual and ideal body size [9, 14, 15]. Body image discrepancy is identified as one possible reason underlying body dissatisfaction among adolescents [14]. Because girls have more body image concerns than boys [13], understanding factors uniquely related to girls’ body image discrepancy may be important for developing targeted interventions to address body-related issues and prevent adverse health-related sequelae in this population [3, 16].

A few studies were found that examined relationships between demographic factors, such as socioeconomic status (SES) and body image. Some indicated that adolescents of higher SES had greater body image discrepancy than those of middle or lower SES [17], whereas other noted that lower SES was related to greater body image discrepancy [18] and more unhealthy weight control behaviors [19]. In contrast, Story and colleagues [20] reported that higher SES was related to greater weight satisfaction and lower unhealthy weight control behaviors. Others noted that higher SES was associated with eating disorders [21] or showed no relationship between SES and disordered eating attitudes or behaviors [22]. Interestingly, O’Dea [23] found no differences between Australian adolescent girls of low and middle-high SES regarding body image; but, in a later study, O’Dea and Caputi [24] noted girls of low SES were more likely to report being “too thin,” as compared to their middle-high SES counterparts [24]. The ambiguous findings indicate a need for continued research.

Findings concerning race or ethnicity and body image were also equivocal. Some researchers noted no racial or ethnic differences in body dissatisfaction among girls [18, 25]. In contrast, others found that Asian and Hispanic girls had the highest levels of body dissatisfaction with African American girls being less likely to express body dissatisfaction than White girls [5, 10]. In another study, White 5th grade students had lower body image discrepancy than Latino 5th graders with neither group differing from African Americans who were in the same academic grade [15, 26]. In addition, the 5th graders’ body image discrepancy was significantly and positively associated with their body mass index (BMI) [26]. Due to the limited number of studies examining the relationship between BMI (especially when objectively measured) and body image in large samples [27] of racially and ethnically diverse girls, consensus indicating that those who are overweight or obese experience greater body image discrepancy or satisfaction/dissatisfaction than those who have a healthy weight has not yet been reached [28]. No studies were found that specifically examined the relationship between girls’ body image discrepancy or satisfaction/dissatisfaction and percent body fat.

Besides demographics, certain behaviors may be related to body image. Schneider and colleagues [18] reported that more time spent watching television (TV) daily was related to a higher level of body dissatisfaction among adolescent girls, whereas Añez and colleagues [29] found no association. Interestingly, in the latter study, adolescent girls’ greater number of hours of computer use for leisure was related to higher body dissatisfaction, but a higher number of hours using the computer for homework was associated with lower body dissatisfaction. The researchers hypothesized that web-based surfing or social networking during leisure time may have exposed the girls to information about the thin-beauty ideal, which is promoted extensively yet unattainable for most. Girls’ internalization of the thin-beauty ideal may have resulted in negative body image perceptions [29]. Although this contention is plausible, without information about the type of material viewed in the media, interpreting any findings is difficult.

Several studies have suggested that increased physical activity (PA) participation is associated with a more positive body image among adolescent girls. Unfortunately, findings were limited in that PA participation was either self-reported by the girls [29, 30] or based only on whether girls participated in sports [18] or dance [31, 32]. Altıntaş and colleagues [33] found that self-reported PA was not correlated with body image satisfaction among adolescent girls. No study was found that included an objective measure of girls’ PA.

Three studies including adolescents were found that examined body image and cardiovascular (CV) fitness. In the single cross-sectional study, increased fitness was associated with a more positive body image among 8- to 16-year-olds [28]. In the remaining two, both of which were randomized controlled trials with overweight and obese adolescents, body image improved following participation in the PA intervention offered in each study [34, 35]. Whether these findings apply to girls is unknown because results were not presented separately for each sex in any of the three studies.

To address some ambiguous findings noted in the literature regarding this area of research, the aims of this study were to: 1) examine demographic (e.g., age, grade, ethnicity, race, and enrollment in the free or reduced-price lunch program) differences in body image discrepancy; and 2) determine the association of body image discrepancy with weight status, percent body fat, PA, sedentary behavior, and CV fitness among young adolescent girls in the U.S. This study makes an important contribution by including a large sample of girls of minority status and objective measures of their height and weight, percent body fat, PA, and CV fitness.

## Methods

### Design, participants, and setting

The study is a secondary cross-sectional analysis of baseline data from a large group randomized controlled trial (RCT) including girls in 5th to 8th grade in 24 Midwestern U.S. schools. In the RCT, eight schools per year over 3 years (2012–2015) were randomly assigned to either receive a multi-component PA intervention called “Girls on the Move” or serve as a control condition. Additional information regarding the trial and related inclusion and exclusion criteria have been published previously [36]. Baseline data collected during the fall of 2012, 2013, and 2014 were used for this study.

### Measures

#### Demographics

Five questions, including age, grade, ethnicity, race, and enrollment in the free or reduced-price lunch program, listed in the consent form were completed by girls’ parents or guardians in collaboration with their daughters if needed.

#### Body image discrepancy

The Contour Drawing Rating Scale was used to assess girls’ perceived current/actual and ideal body image [37]. The scale includes nine female front-view drawings of precisely graduated size silhouettes, rated from 1 (severely underweight; thinnest) to 9 (very obese; largest) [2]. Girls selected one of the numbers to respond to each of the following two questions: “Which figure do you think looks the most like you now?” and “Which figure looks like how you want to look?” The scale has been used with girls in early adolescence and has been shown to have good test-retest reliability ranging from .65 to .87 and acceptable construct validity with moderate correlation (*r* = .64) between current/actual body image and measured BMI [38]. Body image discrepancy was measured by computing the difference between current/actual and ideal body image ratings (current/actual – ideal rating). A higher degree of incongruence between the number selected to represent current/actual and the one chosen for ideal body image indicates greater body image discrepancy. For example, a difference score of zero indicates complete satisfaction with one’s body. On the other hand, scores range between −8 and −1 for girls who want a larger figure range and between 1 and 8 for those who prefer a thinner figure [2].

#### BMI and percent body fat

Girls’ BMI was calculated from height and weight (weight in kg/height in m^2^) measured behind a privacy screen. Height was measured to the nearest .10 cm with a Shorr Board (Weigh and Measure, LLC, Olney, MD). After a data collector entered a girl’s age, sex, and height into a portable digital foot-to-foot bioelectric impedance scale, the girl stood still over the center of the scale with body weight evenly distributed over the feet, feet next to each another, and arms hanging freely by each side of the body. Each girl wore lightweight clothing (e.g., removed heavy jewelry, belts, coins, cell phones, glasses and sweatshirts/sweaters) and was barefoot. Both percent body fat estimated to the nearest 0.1% and weight measured to the nearest .10 kg were obtained with the same scale (Tanita Corporation, Tokyo, Japan). Each girl’s height and weight were measured two times, and the average values were used to calculate BMI (weight in kg/height in meters squared). BMI-percentile (BMI-P) and BMI-z score were estimated, based on the Centers for Disease Control and Prevention online BMI-for–age growth charts. Based on the growth charts, underweight, healthy weight, overweight, and obese were defined as having a BMI-P < 5th, 5th to < 85th, 85th to < 95th, and ≥ 95th, respectively [39].

#### Sedentary behavior (screen time)

Screen time was assessed by six items with six response choices ranging from 0 = *I do not* (the specific behavior) on a day (school or weekend) to 5 = *5 or more hours per day*. Girls’ responses to the items were analyzed to identify the number of hours per day that girls spent viewing TV or movies, playing video games or using the computer or web (Internet) for something that was not schoolwork, and talking on the phone or sending messages on a typical school day (3 items total) and weekend day (3 items total). Items were adapted from those listed in Middle School Youth Risk Behavior Surveys [40]. Cronbach’s alpha was .81 with item-total correlation coefficients ranging from .49 to .64.

#### Self-reported physical activity

To assess moderate-to-vigorous PA (MVPA), girls responded to a PA Screening Measure including two items asking about the number of days per week that they had participated in physical activities for 60 min or more, during which they started to sweat and experienced an increase in heart and breathing rate. One item elicited the number of days during the last 7 days, and the other focused on the number of days during a typical or average week. MVPA was determined by calculating the average of the two responses. The measure has been found to be reliable, and moderate correlations with accelerometer data have been reported [41]. In this study, self-reported MVPA score ranged from 0 to 7, with a positive correlation with accelerometer-measured MVPA (*r* = .16, *p* < .001).

#### Accelerometer-measured physical activity

Minutes of PA were assessed via the Actigraph GT3X-plus accelerometer (actigraphcorp.com), which is reliable and valid for measuring this behavior [42, 43]. Girls were asked to wear the monitor attached to an elastic belt on their right hip for 7 days from the time arising in the morning to bedtime, except when swimming or bathing. Minutes engaged in various activity intensities were estimated based on the following cut-points for recorded acceleration counts: sedentary activity ≤ 25 counts/15 s; light PA 26–573 counts/15 s; moderate PA 574–1002 counts/15 s, and vigorous PA ≥ 1003 counts/15 s [44, 45]. Additional details concerning the procedure are available elsewhere [36].

#### CV fitness

The Progressive Aerobic Cardiovascular Endurance Run (PACER) test, an endurance shuttle run, was employed to estimate girls’ CV fitness [46]. Each girl ran 15 m or 20 m laps (depending on school space) between two cones until she could no longer continue, trying to reach each cone before hearing an audio cue. As the PACER test progressed, a decrease in the time between the audio cues occurred, requiring girls to increase the pace of their running in order to reach the next cone in time. The test ended for a girl when she was unable to reach the next cone during two different laps by the time the audio cue had sounded. Each girl’s number of laps was converted to the mile-equivalent and used to estimate VO_2max_ (ml/kg/min) based on the following equation: VO_2_ = (−8.41*(mile-equivalent)) + (0.34*(mile-equivalent*mile-equivalent)) + (0.21*(age*gender)) - (0.84*body mass index) + 108.94 [47]. A higher VO_2max_ value indicates greater CV fitness.

### Procedures

The study was approved by the Michigan State University Institutional Review Board (IRB) and school district administrators. Recruitment procedures have already been reported [36]. Prior to study enrollment and data collection, informed written consent was obtained from parents/guardians to indicate that their daughter had permission to participate, and informed written assent was obtained from the girls. The study’s measurement coordinator trained all data collectors prior to the data collection that occurred at the girls’ schools. During data collection, girls completed an iPad-delivered survey that included the scales for measuring body image discrepancy, MVPA, and screen time. Two data collectors assessed each girl’s weight and estimated her percent body fat via a portable digital foot-to-foot bioelectric impedance scale. A Shorr board was used to measure each girl’s height. On the same day, as soon as each girl completed the measures, she was asked to join a small group with about 5 other girls to begin the PACER test. Two other data collectors observed one small group at a time engaging in the PACER test, which was set up in a large room in the girls’ school [46]. The data collectors counted and recorded the number of laps that each girl was able to successfully complete during the test. PACER test recommendations were followed to estimate each girl’s CV fitness from her recorded number of laps. Finally, girls received verbal and written information about the accelerometer and received an accelerometer to wear. Additional procedural information is described elsewhere [36].

### Statistical analyses

SPSS Statistics 22 was used to analyze the study data. Descriptive statistics, including means, standard deviations, frequency, percentage, were used to describe study variables. Independent t-test, one-way ANOVA, or Pearson correlations were applied to examine demographic differences and relationships between body image discrepancy and other variables, such as screen time, PA, weight, and CV fitness. Two-way ANOVA was performed to examine the interaction effect of race and weight status on body image discrepancy. Partial correlation and hierarchical linear regressions were applied to explore the possible correlates of body image discrepancy after controlling for demographic characteristics.

## Results

### Sample characteristics

Table 1 demonstrates the participant characteristics. A total of 1519 girls participated, with a mean age of 12.05 and a range from 10 to 15 years old. Nearly half (*n* = 756, 49.8%) were Black, 27.1% (*n* = 412) were White, 14% (*n* = 201) were Hispanic, and the majority (*n* = 1182, 83.5%) were enrolled in the school free or reduced-price lunch program. Slightly greater than half were overweight or obese (*n* = 783, 51.7%).

**Table 1:** Demographic characteristics among participants (*N* = 1519)

Variable	Mean	SD
Age	12.05	1.01
	**Frequency (** ***n*** **)**	**Percentage**
Grade		
5th	228	15.0
6th	584	38.4
7th	573	37.7
8th	134	8.8
Hispanic^a^	201	14.0
Race		
White	412	27.1
Black	756	49.8
Other or Mixed-Race	351	23.1
Enrolled in free or reduced-price lunch program^b^	1182	83.5
Weight Status^c^		
Underweight	44	2.9
Healthy Weight	687	45.4
Overweight	305	20.1
Obese	478	31.6

^a^86 missing

^b^52 missing

^c^5 missing

### Body image discrepancy

There was moderate correlation between girls’ current/actual and ideal body image (*r* = .45, *p* < .001). The body image discrepancy mean (current/actual – ideal rating) was 1.10 (*SD* = 1.48, min-max = −5 - 7), suggesting girls on average preferred a thinner figure. About 10.8% (*n* = 163) of the girls chose a larger ideal than current/actual figure, 21.7% (*n* = 329) chose a similar ideal and current/actual figure (exactly the same number associated with each figure selected resulting in a rating difference of 0), and the majority (67.5%; *n* = 1023) chose a smaller ideal than current/actual figure. About 41.6% (*n* = 630) had an absolute discrepancy score greater than one.

### Body image discrepancy and demographics

Girls’ body image discrepancy did not vary across ethnicity (*M*_*Hispanic*_ = 1.09 vs. *M*_*non-Hispanic*_ = 1.11, *p* = .826), grade (*p* = .565), or enrollment status in the free or reduced-price school lunch program (*M*_*enrolled*_ = 1.08 vs. *M*_non-enrolled_ = 1.14, *p* = .58). White girls had a higher body image discrepancy mean score than Black girls (1.24 vs. 1.01, *p* = .035). Specifically, 71.6% of White girls wanted to have a smaller body image, compared to 63.6% of Black girls (*p* = .001).

### Body image discrepancy and weight status

As shown in Table 2, which demonstrates the inter-correlations between study variables, girls’ perception of their current/actual body image and body image discrepancy were moderately and positively correlated with their BMI (*r* = .66, *p* < .001), BMI-P (*r* = .59, *p* < .001), and BMI z-score (*r* = .65, *p* < .001); while their ideal body image was weakly and positively correlated with BMI (*r* = .16, *p* < .001), BMI-P (*r* = .05, *p* = .044), and BMI z-score (*r* = .10, *p* < .001). Percent body fat was positively correlated with body image discrepancy (*r* = .58, *p* < .001). Girls’ body image discrepancy differed significantly according to their weight status (*F*_*3,1506*_ = 171.32, *p* < .001). Specific findings were: (a) overweight (*M* = 1.37, *SD* = 1.07) girls had higher body image discrepancy scores than underweight (*M* = 0.61, *SD* = 1.99, *p* = .002) or healthy weight (*M* = 0.35, *SD* = 1.30, *p* < .001) girls; (b) obese (*M* = 2.04, *SD* = 1.28) girls had higher body image discrepancy scores than underweight (*p* < .001) or healthy weight (*p* < .001) girls; (c) obese girls had higher body image discrepancy than overweight girls (*p* < .001); but (d) no differences occurred between underweight and healthy weight girls (*p* = 1.00). Results from the two-way ANOVA showed no significant interaction effect of race and weight status on body image discrepancy (*F*_*6,1498*_ = 0.58, *p* = .744).

**Table 2:** Inter-correlations between study variables

Variable	1	2	3	4	5	6	7	8	9	10	11	12	13
1. Age	–												
2. BMI	.25^**^	–											
3. BMI-P	.09^**^	.78^**^	–										
4. BMI z-score	.10^**^	.90^**^	.96^**^	–									
5. Percent body fat	.19^**^	.97^**^	.86^**^	.95^**^	–								
6. Screen time	.26^**^	.10^**^	.07^*^	.07^**^	.09^**^	–							
7. Self-reported MVPA	.01	−.05	−.03	−.04	−.05^*^	−.08^**^	–						
8. Accelerometer-measured LPA	−.24^**^	.08^**^	.10^**^	.12^**^	.10^**^	−.14^**^	.06^*^	–					
9. Accelerometer-measured MPA	−.08^**^	−.03	−.02	−.01	−.03	−.12^**^	.15^**^	.57^**^	–				
10. Accelerometer-measured VPA	−.12^**^	−.18^**^	−.16^**^	−.17^**^	−.19^**^	−.12^**^	.14^**^	.26^**^	.65^**^	–			
11. CV fitness	−.25^**^	−.99^**^	−.79^**^	−.91^**^	−.97^**^	−.11^**^	.06^*^	−.08^**^	.03	.19^**^	–		
12. Current/actual body image	.15^**^	.66^**^	.59^**^	.65^**^	.67^**^	.11^**^	−.06^*^	.03	−.04	−.15^**^	−.66^**^	–	
13. Ideal body image	.12^**^	.16^**^	.05^*^	.10^**^	.13^**^	.11^**^	−.01	−.02	−.01	−.01	−.15^**^	.45^**^	–
14. Body image discrepancy	.05^*^	.56^**^	.57^**^	.59^**^	.58^**^	.02	−.06^*^	.05	−.04	−.14^**^	−.55^**^	.66^**^	−.37^**^

*BMI* body mass index, *BMI-P* BMI-percentile, *MVPA* moderate-to-vigorous physical activity, *LPA* light physical activity, *MPA* moderate physical activity, *VPA* vigorous physical activity, *CV* cardiovascular

^*^ *p* < .05, ^**^ *p* < .01

### Body image discrepancy and health behaviors

Girls reported participating in 5.80 (*SD* = 3.46) hours/day of screen time on weekdays and 6.67 (*SD* = 3.61) hours/day on weekend days. No relationship was noted between girls’ self-reported screen time and body image discrepancy (see Table 2). Only 9.2% (*n* = 140) of girls reported participating in the recommended 60 min MVPA for 7 days in a usual 7 days, and 7.0% (*n* = 107) engaged in at least 60 min MVPA for 7 days during the past 7 days. Girls’ self-reported MVPA in a usual 7 days and during the past 7 days was weakly correlated with their body image discrepancy (*r* = −.06, *p* = .033; *r* = −.05, *p* = .035, respectively). The effect was no longer significant after adjusting for race and BMI.

Based on the accelerometer data, girls participated in 39.78 min/h of sedentary activity (*SD* = 18.30), 17.84 min/h of light PA (*SD* = 3.58), 2.09 min/h of moderate PA (*SD* = 0.80), and .74 min/h of vigorous PA (*SD* = 0.53). Only 1.3% (*n* = 20) of girls did not have valid accelerometer data of at least 8 h per day for three weekdays and one weekend day [48]. Both MVPA and vigorous PA were negatively correlated with body image discrepancy (*r* = −.10, *p* < .001; *r* = −.14, *p* < .001, respectively), but the correlations were not significant after adjusting for race and BMI (*r* = −.04, *p* = .168; *r* = −.05, *p* = .056, respectively).

### Body image discrepancy and CV fitness

Girls’ mean CV fitness level, measured by VO_2max_, was 37.89 ml/kg/min (*SD* = 5.19; min-max = 6.72–55.78), which is below the FITNESSGRAM® standards or Healthy Fitness Zone cut-off score of 40.2 for a 10-year-old girl (minimum level of performance that must be achieved before girl of this age is identified as being fit, healthy, or at a reduced risk) [49]. Girls’ body image discrepancy was moderately correlated with their CV fitness (*r* = −.55, *p* < .001). The negative correlation between body image discrepancy and CV fitness weakened after controlling for race and percent body fat (*r* = −.06, *p* = .017).

Table 3 shows the results from hierarchical regression models. After adjusting for demographics (academic grade, Hispanic ethnicity, race, and free or reduced-price school lunch program), percent body fat, but not CV fitness or MVPA, significantly influenced girls’ body image discrepancy. Girls with higher percent body fat had a higher body image discrepancy score. The whole model explained about 34% of the variance in body image discrepancy. In the 2nd model, girls’ body image discrepancy score decreased by girls’ grade; and White girls had a higher body image discrepancy score than Black girls, showing some suppression effects. These results may be due to girls’ grade being negatively correlated with their accelerometer-measured MVPA (*r* = −.10, *p* < .001) and CV fitness (*r* = −.25, *p* < .001), but positively correlated with percent body fat (*r* = .19, *p* < .001). Moreover, Black girls participated in higher accelerometer-measured MVPA (2.96 vs. 2.67, *p* < .001), but had lower CV fitness (37.32 vs. 38.83, *p* < .001) and percent body fat (31.00 vs. 28.17, *p* < .001) than White girls. Further analysis with adding one variable at a time to the demographic model indicated that percent body fat was the common suppressor variable between body image discrepancy, grade and race.

**Table 3:** Hierarchical linear regression of correlates of body image discrepancy (*N* = 1308)

Variable	B (95% CI)	SE	*p*-value	R^2^
Step 1				.002
Grade	0.06 (−0.04, 0.16)	0.05	.214	
Hispanic	−0.05 (−0.30, 0.20)	0.13	.684	
Race	0.06 (−0.03, 0.18)	0.05	.184	
School lunch program	−0.06 (−0.28, 0.16)	0.11	.609	
Step 2				.34
Grade	−0.13 (−0.21, −0.04)	0.04	.003	
Hispanic	−0.12 (−0.32, 0.08)	0.10	.234	
Race	0.15 (0.07, 0.24)	0.04	.001	
School lunch program	−0.04 (−0.22, 0.14)	0.09	.669	
Percent body fat	0.11 (0.08, 0.14)	0.01	<.001	
MVPA	−0.03 (−0.08, 0.03)	0.03	.363	
Cardiovascular fitness	0.04 (−0.01, 0.09)	0.03	.143	

Hispanic: 0 = non-Hispanic, 1 = Hispanic; Race: 1 = Black, 2 = White, 3 = Other or mixed-race; School lunch program: 0 = no, 1 = yes

## Discussion

Overall, we found that 67.5% of the 10- to 15-year-old girls chose smaller ideal body images than their selected current/actual figure. Other studies listed the following specific percentages of girls who wished they were smaller or thinner than their current/actual body image: 73.0% (*n* = 106) of 14- to 17-year-olds in Germany [18], 48.7% (*n* = 178) of 12- to 15-year-olds in South Korea [50], 58.6% (*n* = 188) of 11- to 15-year-olds in South Africa [51], and 32.8% (*n* = 79) of 12- to 15-year-olds in the U.S [50]. The high percentage noted in this study, as well some of the others, underscores an urgent need to address the problem.

With regard to race, the finding that Black girls perceived less body image discrepancy or lower body dissatisfaction than White girls is consistent with results from previous research [5, 10, 13]. In one study involving girls, identifying as being Black in high school predicted a lower increase in body dissatisfaction post-high school 5 years later. The researchers conducting the latter study explained that a large ideal body size may be consistent with the natural shape of Black girls as they progress through high school and beyond. Consequently, the departure from the ideal body shape is likely to be less, resulting in more protection against rising body dissatisfaction [12]. Health professionals may need to consider racial differences when counseling Black girls on the benefits of certain healthy behaviors, such as PA, because focusing on how engagement in them can assist with attaining or maintaining a healthy weight may hold less relevance for this group than for White girls who experience higher body image discrepancy. Discussing perceived benefits of PA specifically identified by Black girls, such as the behavior helps with “staying in shape,” may be a more fruitful approach for promoting positive behavior change [52].

Consistent with findings of Kelly et al. [10] and Monteiro et al. [32], SES was not correlated with either body satisfaction or dissatisfaction among adolescent girls, respectively. However, other researchers reported that lower SES among girls of high school age correlated with greater body dissatisfaction [12] and body discrepancy [18]. Paxton et al. [12] suggested that low self-esteem among high school girls of low SES, possibly resulting from a reduced ability to afford fashionable clothing, may negatively impact girls’ view of their bodies. In this study, the girls had not yet reached high school and a high percentage were of low SES, both of which may have contributed to the unexpected results.

Aligned with the findings of this study, accumulating research indicates that higher BMI is associated with lower body satisfaction [13, 33], a more negative body image [30], and greater discrepancy between their current/actual and ideal body image [25] with obese girls being the least likely to report high body satisfaction [10]. For both junior high and high school girls, BMI emerged as a significant positive predictor of body dissatisfaction 5 years later when the girls were in high school and post-high school, respectively [12]. In efforts to promote positive body-related perceptions among adolescent girls, BMI remains an important factor to consider [53]. Based on findings that overweight adolescent girls having very low body satisfaction had a nearly three unit greater increase in BMI over a 10-year period than those with high body satisfaction, Loth and colleagues [11] suggested that health professionals working with overweight adolescent girls should direct some effort toward promoting a positive body image.

In this study, negative correlations with body image discrepancy were very low for MVPA and moderate for CV fitness, but associations were not significant any more after adjusting for demographics and percent body fat. These suppressions effects may imply that percent body fat has a direct relationship with body image discrepancy, while PA and CV fitness have an indirect effect with body image discrepancy via percent body fat. The underlying causes for the suppression effects warrant further investigation. Therefore, assisting girls to attain and sustain a healthy weight remains critical for helping them to achieve and maintain a positive body image. The high negative correlation between CV fitness and percent body fat indicates that interventions to promote a positive body image among girls may need to include strategies to increase PA as a means to improve their CV fitness, thereby reducing their percent body fat. Evidence has supported that the increased muscle tone, strength, physical competence or fitness, and reduced body size, occurring from increased PA can help to improve body image perceptions [54, 55]. Burgess, Grogan, and Burwitz [31] found that participation in 6 weeks of aerobic dance significantly reduced adolescent girls’ body dissatisfaction. In another study, the BMI of girls who were not involved in dance practice was positively correlated with body dissatisfaction; however, for those practicing dance, the same significant relationship did not occur [32]. According to Kelly [10], girls with high body satisfaction are more likely to report exercising and being fit than those with low body satisfaction. Although PA and CV fitness may have a positive effect on adolescent girls’ body image, one challenge that needs to be overcome is that adolescent girls who are overweight or obese, especially those who have body-related concerns, are likely to avoid attaining PA [3] at an intensity sufficient enough to improve their CV fitness.

This study’s findings showing no relationship between self-reported screen time and body image discrepancy are different from those of Schneider et al. [18] and Añez et al. [29] who found that time spent watching TV [18] and leisure time computer use [29] were associated with a more negative body image, respectively. Añez and colleagues [29] hypothesized that girls’ exposure to the thin-beauty ideal during leisure-time computer use might negatively affect their body image perception [29]. Based on this contention, the null findings in this study may have resulted from no-to-minimal exposure or limited influence from any exposure to the thin-beauty ideal during the girls’ leisure screen time due to the girls being younger than those in the other two studies.

### Strengths and limitations

This study had strengths and limitations. Strengths included a large sample of girls of minority status. A second strength was the use of objective measures to estimate BMI, PA, percent body fat, and CV fitness. Limitations included the self-reported screen time and cross-sectional design, the latter of which prevented the determination of causality or differentiation of a precursor from a consequence of body image discrepancy. The omission of a measure of body distortion or other scales measuring body satisfaction/dissatisfaction limited the comprehensiveness of the study. Limitations were also related to the use of a figural drawing scale to assess body image. As noted by Gardner and Brown [56], use of a limited number of response choices in figural drawing scales may not be sufficient for representing a near-continuous variable. In addition, test-retest reliability may be inflated among adolescents because the vast majority of their responses are usually selected from a small subset of the scale. Moreover, representations of the human form may be unrealistic or inappropriate for certain racial or ethnic groups. Finally, a biased response may result when figures are presented in ascending sizes from left to right and not in a random order. Due to the existing suppression effects, careful consideration is needed when interpreting the pairwise correlations between body image discrepancy and other study variables.

## Conclusion

This study provided important information on factors related to body image discrepancy among young adolescent girls. To our knowledge, it was the first to examine relationships between body image discrepancy and objectively measured PA, CV fitness and percent body fat in this population. A need exists for longitudinal studies to determine whether improving body composition and CV fitness results in a decrease in body image discrepancy among girls. Testing strategies to promote the development of a positive body image is important for preventing the development of physical and mental health conditions, such as eating disorders and low self-esteem. Information from this study may be used to inform screening practices and direct prevention efforts toward groups at high risk for body image discrepancy.

## Abbreviations

ANOVA: Analysis of variance
BMI: Body mass index
BMI-P: Body mass index-percentile
CV: Cardiovascular
IRB: Institutional review board
M: Mean
MD: Maryland
MVPA: Moderate to vigorous physical activity
PA: Physical activity
PACER: Progressive aerobic cardiovascular endurance run
RCT: Randomized controlled trial
SD: Standard deviation
SES: Socioeconomic status
U.S.: United States

## References

[1] Kann L, McManus T, Harris WA, Shanklin SL, Flint KH, Hawkins J (2016). Youth risk behavior surveillance — United States, 2015. MMWR Surveill Summ.

[2] Duchesne AP, Dion J, Lalande D, Bégin C, Émond C, Lalande G, et al. Body dissatisfaction and psychological distress in adolescents: is self-esteem a mediator? J Health Psychol. 2016; doi: 10.1177/1359105316631196. 10.1177/1359105316631196 26929171

[3] Voelker DK, Reel JJ, Greenleaf C (2015). Weight status and body image perceptions in adolescents: current perspectives. Adolesc Health Med Ther.

[4] Bearman SK, Martinez E, Stice E, Presnell K (2006). The skinny on body dissatisfaction: a longitudinal study of adolescent girls and boys. J Youth Adolesc..

[5] Bucchianeri MM, Fernandes N, Loth K, Hannan PJ, Eisenberg ME, Neumark-Sztainer D (2016). Body dissatisfaction: do associations with disordered eating and psychological well-being differ across race/ethnicity in adolescent girls and boys?. Cultur Divers Ethnic Minor Psychol.

[6] Neumark-Sztainer D, Bauer KW, Friend S, Hannan PJ, Story M, Berge JM (2010). Family weight talk and dieting: how much do they matter for body dissatisfaction and disordered eating behaviors in adolescent girls?. J Adolesc Health.

[7] Klaczynski PA (2004). A dual-process model of adolescent development: implications for decision making, reasoning, and identity. Adv Child Dev Behav.

[8] Cash TF, Cash TF, Pruzinsky T (1990). The psychology of physical appearance: aesthetics, attributes, and images. Body images: development, deviance and change.

[9] Thompson JK, Heinberg LJ, Altabe M, Tantleff-Dunn S (1999). Exacting beauty: theory, assessment, and treatment of body image disturbance.

[10] Kelly AM, Wall M, Eisenberg ME, Story M, Neumark-Sztainer D (2005). Adolescent girls with high body satisfaction: who are they and what can they teach us?. J Adolesc Health.

[11] Loth KA, Watts AW, van den Berg P, Neumark-Sztainer D (2015). Does body satisfaction help or harm overweight teens? A 10-year longitudinal study of the relationship between body satisfaction and body mass index. J Adolesc Health.

[12] Paxton SJ, Eisenberg ME, Neumark-Sztainer D (2006). Prospective predictors of body dissatisfaction in adolescent girls and boys: a five-year longitudinal study. Dev Psychol.

[13] van den Berg PA, Mond J, Eisenberg M, Ackard D, Neumark-Sztainer D (2010). The link between body dissatisfaction and self-esteem in adolescents: similarities across gender, age, weight status, race/ethnicity, and socioeconomic status. J Adolesc Health.

[14] Gilliland MJ, Windle M, Grunbaum JA, Yancey A, Hoelscher D, Tortolero SR (2007). Body image and children's mental health related behaviors: results from the healthy passages study. J Pediatr Psychol.

[15] Michael SL, Wentzel K, Elliott MN, Dittus PJ, Kanouse DE, Wallander JL (2014). Parental and peer factors associated with body image discrepancy among fifth-grade boys and girls. J Youth Adolesc.

[16] Ata RN, Ludden AB, Lally MM (2007). The effects of gender and family, friend, and media influences on eating behaviors and body image during adolescence. J Youth Adolesc.

[17] Wang Z, Byrne NM, Kenardy JA, Hills AP (2005). Influences of ethnicity and socioeconomic status on the body dissatisfaction and eating behaviour of Australian children and adolescents. Eating Behav.

[18] Schneider S, Weiss M, Thiel A, Werner A, Mayer J, Hoffmann H (2013). Body dissatisfaction in female adolescents: extent and correlates. Eur J Pediatr.

[19] Neumark-Sztainer D, Story M, Falkner NH, Beuhring T, Resnick MD (1999). Sociodemographic and personal characteristics of adolescents engaged in weight loss and weight/muscle gain behaviors: who is doing what?. Prev Med.

[20] Story M, French SA, Resnick MD, Blum RW (1995). Ethnic/racial and socioeconomic differences in dieting behaviors and body image perceptions in adolescents. Int J Eat Disord.

[21] Walters EE, Kendler KS (1995). Anorexia nervosa and anorexic-like syndromes in a population-based female twin sample. Am J Psychiatry.

[22] Deleel ML, Hughes TL, Miller JA, Hipwell A, Theodore LA (2009). Prevalence of eating disturbance and body image dissatisfaction in young girls: an examination of the variance across racial and socioeconomic groups. Psychol Sch.

[23] O’Dea J (1994). Food habits, body image and self-esteem of adolescent girls from disadvantaged and non-disadvantaged backgrounds. Australian. J Nutr Diet.

[24] O'Dea JA, Caputi P (2001). Association between socioeconomic status, weight, age and gender, and the body image and weight control practices of 6- to 19-year-old children and adolescents. Health Educ Res.

[25] Saunders JF, Frazier LD. Body dissatisfaction in early adolescence: the coactive roles of cognitive and sociocultural factors. J Youth Adolesc. 2016; doi: 10.1007/s10964-016-0559-2. 10.1007/s10964-016-0559-2 27619380

[26] Wallander JL, Taylor WC, Grunbaum JA, Franklin FA, Harrison GG, Kelder SH (2009). Weight status, quality of life, and self-concept in African American, Hispanic, and white fifth-grade children. Obesity (Silver Spring).

[27] ter Bogt TF, van Dorsselaer SA, Monshouwer K, Verdurmen JE, Engels RC, Vollebergh WA (2006). Body mass index and body weight perception as risk factors for internalizing and externalizing problem behavior among adolescents. J Adolesc Health.

[28] Schubert A, Januario RS, Casonatto J, Sonoo CN (2013). Body image, nutritional status, abdominal strength, and cardiorespiratory fitness in children and adolescents practicing sports. Rev Paul Pediatr.

[29] Añez E, Fornieles-Deu A, Fauquet-Ars J, López-Guimerà G, Punti-Vidal J, Sánchez Carracedo D. Body image dissatisfaction, physical activity and screen-time in Spanish adolescents. J Health Psychol. 2016; doi: 10.1177/1359105316664134. 10.1177/1359105316664134 27557652

[30] Kantanista A, Osiński W, Borowiec J, Tomczak M, Król-Zielińska M (2015). Body image, BMI, and physical activity in girls and boys aged 14-16 years. Body Image.

[31] Burgess G, Grogan S, Burwitz L (2006). Effects of a 6-week aerobic dance intervention on body image and physical self-perceptions in adolescent girls. Body Image.

[32] Monteiro LA, Novaes JS, Santos ML, Fernandes HM (2014). Body dissatisfaction and self-esteem in female students aged 9-15: the effects of age, family income, body mass index levels and dance practice. J Hum Kinet.

[33] Altıntaş A, Aşçı FH, Kin-İşler A, Güven-Karahan B, Kelecek S, Özkan A (2014). The role of physical activity, body mass index and maturity status in body-related perceptions and self-esteem of adolescents. Ann Human Biol.

[34] Goldfield GS, Adamo KB, Rutherford J, Murray M (2012). The effects of aerobic exercise on psychosocial functioning of adolescents who are overweight or obese. J Pediatr Psychol.

[35] Goldfield GS, Kenny GP, Alberga AS, Prud'homme D, Hadjiyannakis S, Gougeon R, Phillips P, Tulloch H, Malcolm J, Doucette S (2015). Effects of aerobic training, resistance training, or both on psychological health in adolescents with obesity: the HEARTY randomized controlled trial. J Consult Clin Psychol.

[36] Robbins LB, Pfeiffer KA, Vermeesch A, Resnicow K, You ZY, An L (2013). “Girls on the Move” intervention protocol for increasing physical activity among low-active underserved urban girls: a group randomized trial. BMC Public Health.

[37] Thompson MA, Gray JJ (1995). Development and validation of a new body-image assessment scale. J Pers Assess.

[38] Wertheim EH, Paxton SJ, Tilgner L (2004). Test-retest reliability and construct validity of contour drawing rating scale scores in a sample of early adolescent girls. Body Image.

[39] Centers for Disease Control and Prevention (2015). About child & teen BMI.

[40] Centers for Disease Control and Prevention (2016). 2009 and 2011 middle school youth risk behavior survey.

[41] Prochaska JJ, Sallis JF, Long B (2001). A physical activity screening measure for use with adolescents in primary care. Arch Pediatr Adolesc Med.

[42] Hänggi JM, Phillips LRS, Rowlands AV (2013). Validation of the GT3X ActiGraph in children and comparison with the GT1M ActiGraph. J Science Med Sport.

[43] Trost SG, Ward DS, Moorehead SM, Watson PD, Riner W, Burke JR (1998). Validity of the computer science and applications (CSA) activity monitor in children. Med Sci Sports Exerc.

[44] Evenson KR, Catellier DJ, Gill K, Ondrak KS, McMurray RG (2008). Calibration of two objective measures of physical activity for children. J Sports Sci.

[45] Trost SG, Loprinzi PD, Moore R, Pfeiffer KA (2011). Comparison of accelerometer cut points for predicting activity intensity in youth. Med Sci Sports Exerc.

[46] Meredith MD, Welk JG, The Cooper Institute (2010). Fitnessgram & activitygram test administration manual.

[47] Cureton KJ, Sloniger MA, O'Bannon JP, Black DM, McCormack WP (1995). A generalized equation for prediction of VO2peak from 1-mile run/walk performance. Med Sci Sports Exerc.

[48] Fitzgibbon ML, Stolley MR, Schiffer L, Kong A, Braunschweig CL, Gomez-Perez SL (2013). Family-based hip-hop to health: outcome results. Obesity (Silver Spring).

[49] Morrow JR, Zhu W, Mahar MT, Plowman SA, Meredith D (2013). Physical fitness standards for children. Fitnessgram/activitygram reference guide.

[50] Jung J, Forbes GB, Lee Y (2009). Body dissatisfaction and disordered eating among early adolescents from Korea and the U.S. Sex Roles.

[51] Pedro TM, Micklesfield LK, Kahn K, Tollman SM, Pettifor JM, Norris SA (2016). Body image satisfaction, eating attitudes and perceptions of female body silhouettes in rural South African adolescents. PLoS One.

[52] Grieser M, MB V, Bedimo-Rung AL, Neumark-Sztainer D, Moody J, Young DR (2006). Physical activity attitudes, preferences, and practices in African American, Hispanic, and Caucasian girls. Health Educ Behav.

[53] Holsen I, Carlson Jones D, Skogbrott Birkeland M (2012). Body image satisfaction among Norwegian adolescents and young adults: a longitudinal study of the influence of interpersonal relationships and BMI. Body Image.

[54] Cash TF, Cash TF, Pruzinsky T (2002). Cognitive behavioral perspectives on body image. Body image: a handbook of theory, research, and clinical practice.

[55] Martin KA, Lichtenberger CM, Cash TF, Pruzinsky T (2002). Fitness enhancement and changes in body image. Body image: a handbook of theory, research, and clinical practice.

[56] Gardner RM, Brown DL (2010). Body image assessment: a review of figural drawing scales. Pers Individ Dif.

